# Transformation of a Malaysian species of *Nannochloropsis*: gateway to construction of transgenic microalgae as vaccine delivery system to aquatic organisms

**DOI:** 10.1080/21655979.2020.1822106

**Published:** 2020-09-29

**Authors:** Aisamuddin Ardi Zainal Abidin, Mohanrajh Suntarajh, Zetty Norhana Balia Yusof

**Affiliations:** a Department of Biochemistry, Faculty of Biotechnology and Biomolecular Sciences, Universiti Putra Malaysia, Serdang, Malaysia; b Aquatic Animal Health and Therapeutics Laboratory (Aquahealth), Institute of Bioscience, Universiti Putra Malaysia, Serdang, Malaysia; c Bioprocessing and Biomanufacturing Research Center, Universiti Putra Malaysia, Serdang, Malaysia

**Keywords:** Aquaculture, electroporation, delivery system, microalgae, *Nannochloropsis* sp., transformation

## Abstract

Nannochloropsis

sp. is a green alga that is widely used in the aquaculture industry as a feed in Malaysia, but genetic engineering studies of this alga are still underexplored even though there is a growing interest in microalgae genetic engineering for various industrial purposes. This study aims to investigate the efficiency of three transformation methods normally done on microalgae, namely polyethylene glycol (PEG), electroporation, and glass beads on Malaysian indigenous *Nannochloropsis* sp. using two commercially available plasmids, pUC19 and pGEM-T easy vector as well as an amplicon of ampicillin resistance (AMPR) gene. In this study, out of three transformation methods tested, positive transformants of *Nannochloropsis* sp. were successfully obtained via electroporation method. Further verification via polymerase chain reaction (PCR) and sequencing confirmed that the electroporation method was found to be the sole successful method in producing transgenic lines of our locally isolated *Nannochloropsis* sp. Results from this study proved the efficiency of electroporation for delivery of transgene to this green alga which has been reported to be tedious. The described method also provides the gateway for developing *Nannochloropsis* sp. as a delivery system to aquatic organism due to its importance in the industry.

## Graphical Abstract

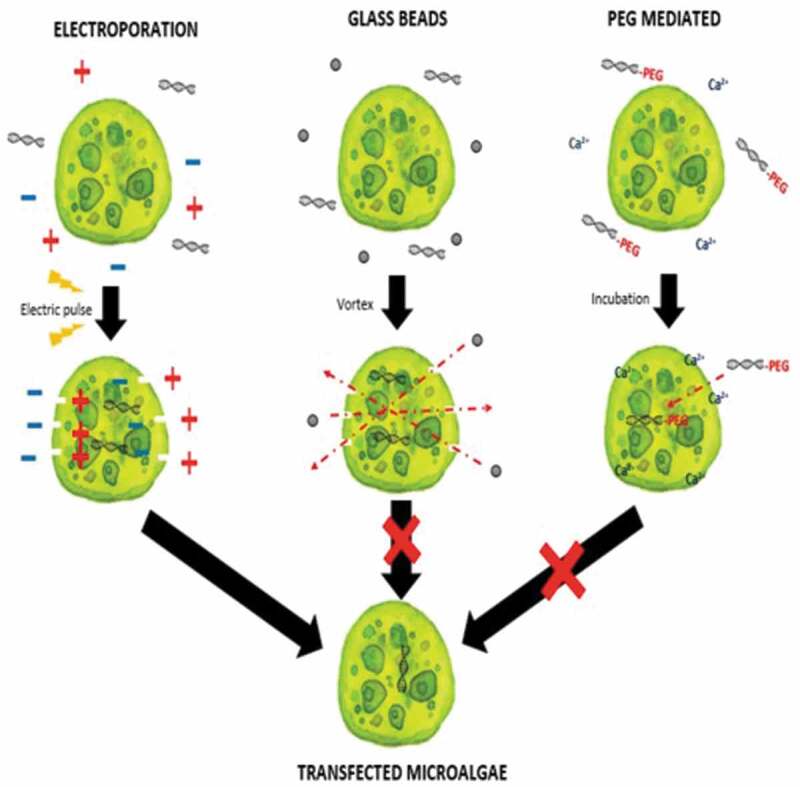

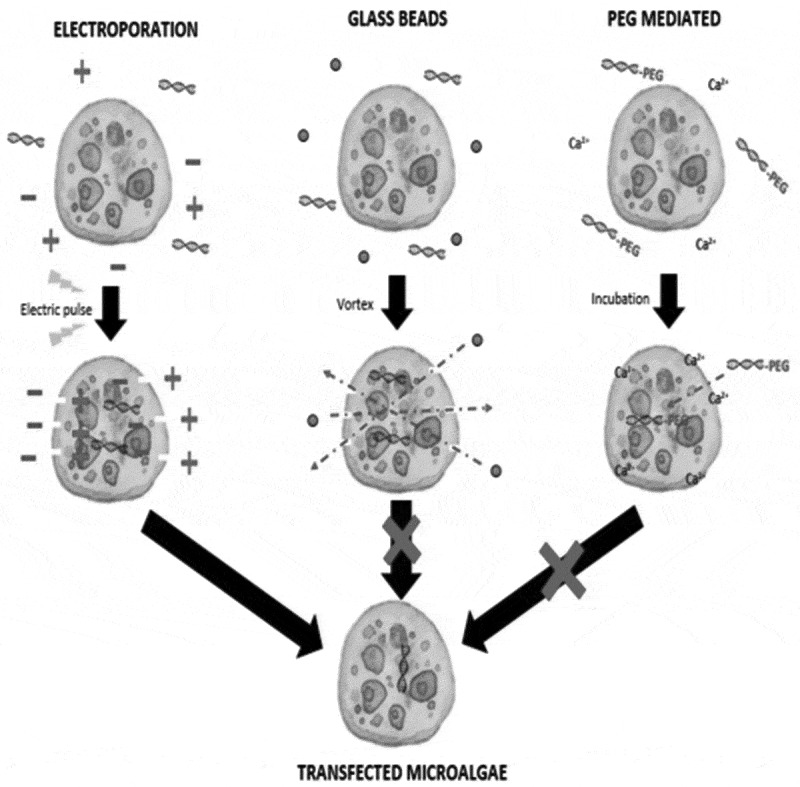

## Introduction

Microalgae are becoming the choice for manufacturing platform for the expression of useful foreign genes due to their rapid growth under photoautotrophic conditions and their economical sustainment compared to other organisms [1]. *Nannochloropsis* sp. is a unicellular photoautotrophic organism which belongs to *Eustigmatophyceae* class [2]. The cell size ranges from 2 to 5 µm and their cell wall thickness range from 10 to 25 nm [3]. They have a simple cell wall which is composed of two components, the ﬁbrillar component, and the amorphous component. Fibrillar made of cellulose, a polymer of 1,4-linked b-D-glucose and the amorphous part consists of proteins, lipids, and polysaccharides [4]. *Nannochloropsis* sp. is majorly utilized in fish hatcheries and also classified as potential microalgae for biodiesel and lipid production [5,6]. Besides that, *Nannochloropsis* sp. has shown high transformation efficiency through homologous recombination, which could lead to useful genomics improvement in alga industry [7]. Currently, green microalgae are used as a biomanufacturing platform for the production of recombinant proteins and small molecules for a range of industries including bioenergy, biopharmaceuticals, biomaterials, nutraceuticals, agriculture and animal health, and cosmetics and personal care [8]. Transgenic microalgae have been produced and currently, transformation is being conducted for future applications such as for human therapeutic protein, oral vaccines, enzyme production, nutritional supplement, enhancing biofuel production, fish growth hormone, and potential antibiotic substitutes [9]. Varieties of methods have been developed for algal transformation such as agitation with the presence of glass beads or silicon carbide whiskers, electroporation, microparticle bombardment, polyethylene glycol (PEG) mediated, and the utilization of vector carrier *Agrobacterium tumifaciens* [10]. However, no methods have been reported on Malaysian *Nannochloropsis* species.

Previous studies have only utilized electroporation as a transforming method for *Nannochloropsis* as shown in Table 1. The drawback of using the electroporation method is it requires an expensive machine in comparison with glass beads and PEG-mediated methods, and not much study have been reported with a stable transformation outcome [11]. Transformation methods are differentiated by its principles in introducing and delivering the genetic materials into the microalgae. Electroporation relies on the emission of electric pulse to create temporary pores in the cells membrane allowing genetic materials to enter the cells. Glass beads method on the other hand is another physical transfection method apart from electroporation which is simpler and inexpensive to create pores in the host cell membrane. PEG-mediated method is a chemical transfection method that enhances permeability of the cell membrane together with calcium ion (Ca^2+^) which mediate the genetic material to enter the host cells. However, glass beads and PEG-mediated methods are often utilized in transforming *Chlorella* species [12] and *Chlamydomonas reinhardtii* [13] instead of *Nannochloropsis* species. Due to the lack of study on Malaysian indigenous species of *Nannochloropsis* transformation, this project focused on optimizing available microalgae transformation methods for *Nannochloropsis* sp. which might enable a more diverse option to transform the microalgae.

**Table 1.** Transformation history of *Nannochloropsis.*

**Recombinant protein**	**Application**	**Transformation method**	**Reference**
Bleomycin resistance protein	Gene annotation study	Electroporation	Vieler et al. [14]
Bleomycin resistance protein	Promoter selection study	Electroporation	Li et al. [15]
Bleomycin resistance protein	Knocking out nitrate reductase gene	Electroporation	Killian et al. [7]
shCP (purple chromoprotein)	New selection marker	Electroporation	Shih et al. [16]
Phleomycin resistance gene	Selective marker study	Electroporation	Ma et al. [17]
Bovine lactoferricin	Fish vaccination	Electroporation	20
Yellowfin porgy [*Acanthopagrus latus]* growth hormone	Functional fish growth hormone	Electroporation	19]

## Materials and methods

### Culture maintenance

*Nannochloropsis* sp. were obtained from Institute Aquaculture and Aquatic Science (I-AQUAS) of UPM, Port Dickson, Negeri Sembilan, Malaysia and maintained in liquid F/2 medium [15] and solid F/2 medium supplemented with 15 g L^−1^ agar and 1.6 g L^−1^ NaHCO_3_ [18]. Cultures were grown in 250-mL conical flask under continuous 24 h light illumination n (120 μmol m^−2^ s^−1^) with a rotary shaker (150 rpm) at 24°C. Cells at early mid growth phase were harvested and subjected to various transformation methods.

### Determination of the minimum inhibitory concentration (MIC) of ampicillin on *Nannochloropsis* sp

Wild type *Nannochloropsis* sp. cultures were spread on plates containing 2, 4, and 6 mg mL^−1^ concentrations of ampicillin in F/2 medium agar and incubated at 24°C under continuous illumination of light. The growth of cells on the plates was visually assessed based on the growth of *Nannochloropsis* sp. after 2 weeks.

### Vector and transgene preparation

**Figure 1.**
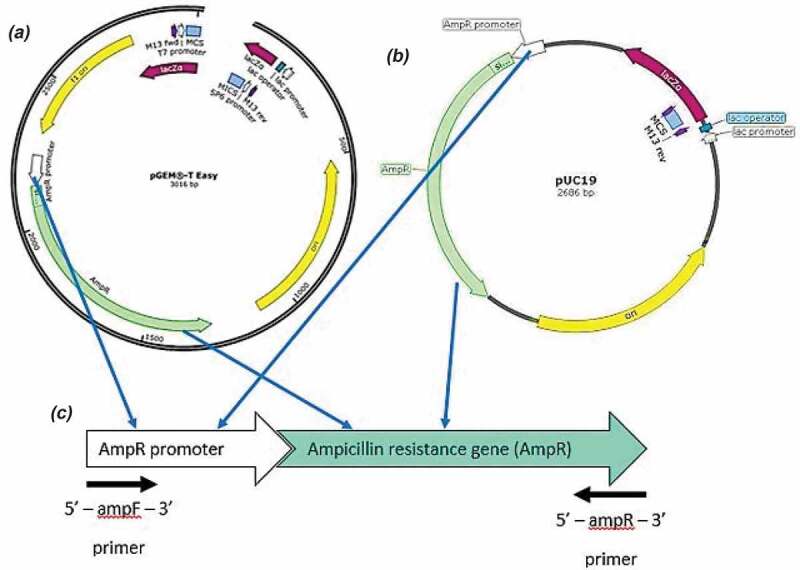
Vectors used in this study which are (A] pGEM-T easy vector, (b) pUC19 vector, and (c) PCR product of the ampicillin resistance gene (AMP^R^).

Two circular cloning vectors, namely pUC19 and pGEM®-T Easy, which harbors AMP^R^ gene commonly used for transformation of bacteria were prepared in two forms, namely circular form and linearized form using *EcoRI* restriction enzyme. AMP^R^ was also amplified from the vector using the following primers: ampF: 5ʹ-CACCGGCTCCAGATTTATCA and ampR: 5ʹ-CCTTCCTGTTTTTGCTCACC-3ʹ (Figure 1). All genetic materials were purified either using innuPREP DNA Mini Kit (Analytik Jena AG) or QIAquick PCR Purification Kit (Qiagen) prior to transformation.

### Preparation *Nannochloropsis* sp. cells

Formation of protoplast (cell wall-less) cells of *Nannochloropsis* sp. was carried out in accordance to Chen et al. [19 and Yang et al. [12, which is described as enzyme-treated cells (cell wall-less) and non-enzyme-treated cells (normal cells). Three milliliters of middle exponential phase cells were collected by centrifugation at 6000 rpm for 2 min at 4°C. The pellet was washed twice with 3 mL of distilled water and then treated with 2 mL enzyme mixture (4% cellulase, 2% macerozyme, and 1% pectinase, in 0.128 M mannitol at pH 5.6) [12]. *Nannochloropsis* sp. cells with enzyme mixture were incubated at 28°C for 8 h in the dark with gentle shaking at 80 rpm [19]. Both enzyme-treated and non-enzyme-treated cells will be further used for the transformation.

### Transformation of *Nannochloropsis* sp

For PEG induction method, only enzyme-treated cells were used and carried out with a similar protocol of Yang et al. [12. Cells were centrifuged at 2500 rpm for 5 min at room temperature, and the pellet was gently suspended in 5 mL of the cultivation medium containing 0.6 M sorbitol and 0.6 M mannitol. The suspension was incubated at room temperature for 5 min and centrifuged again for 5 min. The supernatant was decanted and the pellet was gently resuspended in 5 mL of cultivation medium containing 0.6 M sorbitol and 50 mM CaCl_2_. Then, 10 μg of circular vector DNA was added to 1 mL of enzyme-treated cells. After 15 min of incubation at room temperature, 0.2 mL of PNC solution containing 40% (w/v) PEG 4000, 0.8 M NaCl, 50 mM CaCl_2_ was added with gentle mixing. Following with 30 min of incubation, 600 μL of cultivation medium containing 0.6 M sorbitol, 0.05 M glucose and 1% (w/v) yeast extract was then added and incubated at 28°C in the dark overnight for cell recovery.

Both enzyme-treated and non-enzyme-treated cells were used to transform using the electroporation method with different protocols for each cells. Non-enzyme-treated cells were washed four times at 2500 rpm for 5 min with 384 mM D-sorbitol at 4°C as described by Kilian et al. [7. One milliliter of cells and 10 μg of circular vector DNA were used for each electroporation. Electroporation was performed at 2.2 kV of field strength, 50 μF of capacitance, and 500 Ω of shunt resistance using Bio-Rad Gene Pulser II (Biorad, USA) [7]. Cells were immediately transferred to 15 mL conical Falcon tubes containing 10 mL F/2 medium and incubated in low light overnight. For enzyme-treated cells, electroporation was carried out base on the method of Li et al. [20, with some modifications. 1 mL of enzyme-treated cells were washed twice at 2500 rpm for 5 min with 1 mL of F/2 medium at 4°C. The culture was gently resuspended in 5 mL F/2 medium containing 0.6 M sorbitol and 0.6 M mannitol, and then 10 μg of circular vector DNA was added. After incubation on ice for 10 min, the foreign DNA was introduced into the cells via electroporation at 2.2 kV, 50 μF, and 500 Ω. Cells were quickly transferred to a 15 mL Falcon tube containing 5 mL of F/2 medium and cultured at 28°C for 3–5 days with shaking at 150 rpm.

Glass beads method was carried out based on the method of Feng et al. (2014) [13]. Three milliliters of both non-enzyme-treated and enzyme-treated cells were added with a mixture containing 0.3 g of sterile 106 micron glass beads, 10 μg of circular vector DNA, and 200 μL PNC solution containing 40% (w/v) PEG 4000, 0.8 M NaCl, 50 mM CaCl_2_ in respective glass tubes and agitated at 2000 rpm on a Vortex mixer (Vision Scientific Co., Ltd., Korea) for 1 min. The glass beads were allowed to settle, and cells were transferred to sterilized test tubes containing 2 mL of cultivation medium and cultured under dim light condition for 24 h.

Two hundred microliters of all the regenerated cells at a cell density of approximately 1 × 10^7^ cells mL^−1^ were spread on agar plates containing 6 mg mL^−1^ of ampicillin. Resistant colonies were observed after 2–3 weeks and inoculated into liquid medium after 3–4 weeks.

### Molecular analysis of putative transformants

Liquid cultures of transfected *Nannochloropsis* sp. were grown up to 2 weeks and harvested for verification of transgene incorporation into the genome. Genomic DNA was extracted using DNeasy Plant Mini Kit (Qiagen). The AMP^R^ gene was then amplified using ampF and ampR from the extracted DNA. The PCR products were then further verified via sequencing.

## Results and discussion

### Determination of minimal inhibitory concentration of ampicillin toward *Nannochloropsis* sp

Determining minimal inhibitory concentration is an essential step in developing a transformation protocol. Since microalgae have the natural ability to be resistant to some antibiotics, an antibiotic assay was carried out to determine minimal inhibitory concentration. Thereafter, the concentration will be used for screening transformants, which targeted to kill non-transformed cells and allowing transformed cells to survive. Since the vectors utilized in this study harbor AMP^R^, which confers resistance to the antibiotic ampicillin, the MIC of ampicillin was used for screening. The MIC of ampicillin for *Nannochloropsis* sp. is 6 mg mL^−1^ (Table 2). Ampicillin target the peptidoglycan layer on cell walls of the cells causing a leaky cell, interfering with the cell structure and osmoregulation abilities causing lysis of the prokaryotic and eukaryotic cell. The effect of ampicillin concentrations on the growth of *Nannochloropsis* sp.are as shown in

### Transformation method efficiency for *Nannochloropsis* sp

This study aimed to transform WT *Nannochloropsis* cells and as mentioned previously, there is no well-established method to do so for this particular green microalga. Therefore, a number of transformation methods were explored, namely PEG mediated, electroporation, and glass beads. Positive transformants were obtained for the cells transfected with linear and circular vectors via electroporation of non-enzyme-treated cells. However, for enzyme-treated cells, only linear vector was successfully introduced via electroporation (Table 3).

**Table 2.** Effect of ampicillin on different concentrations on the growth of *Nannochloropsis* sp.

Microalgae species	Effect of ampicillin at different concentrations
*Nannochloropsis* sp.	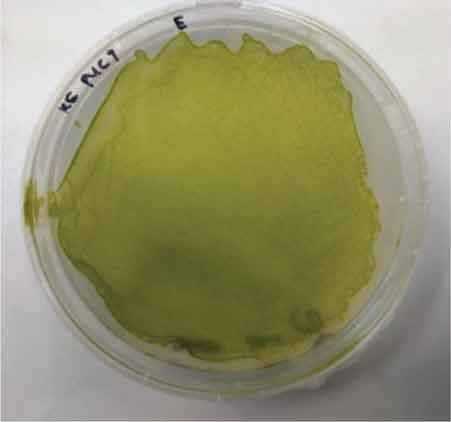 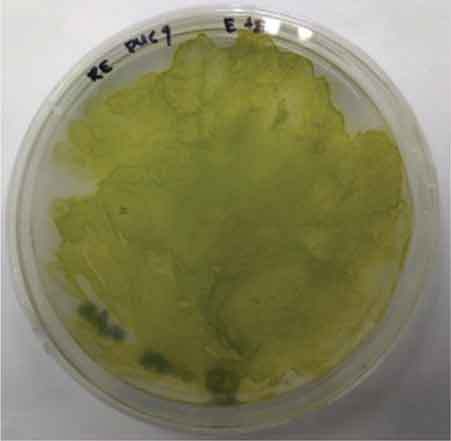 0 mg mL^−1^ 2 mg mL^−1^ 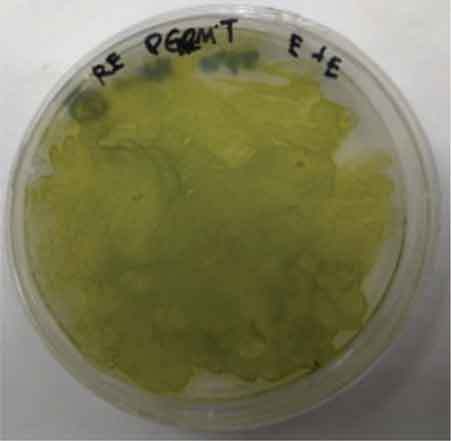 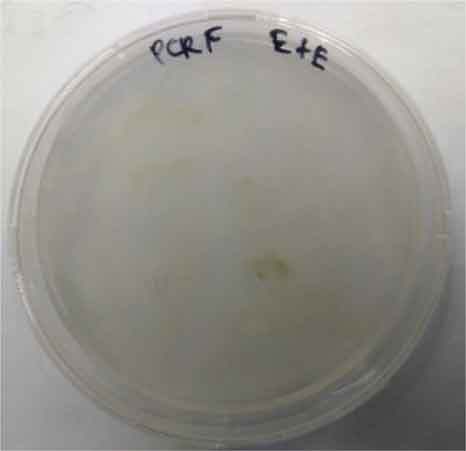 4 mg mL^−1^ 6 mg mL^−1^

**Table 3.** Successful screening of transfected *Nannochloropsis* sp.

	Vector		
Transformation method	pUC19	pGEM®-T Easy	AMP^R^ PCR product
Electroporation with non-enzyme-treated cells	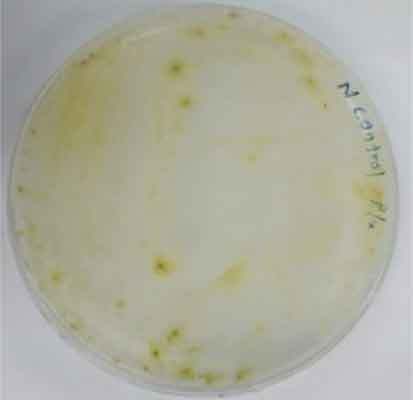 Circular form	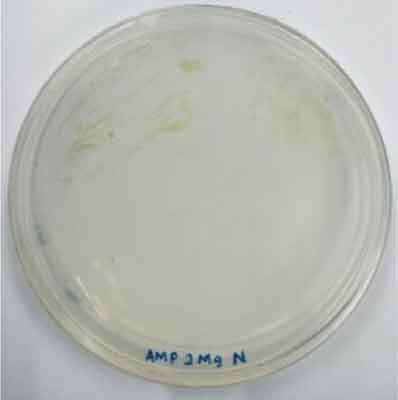 Circular form	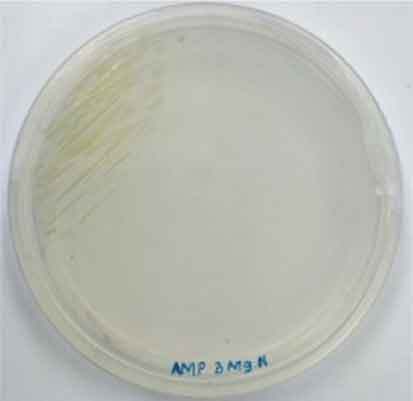
	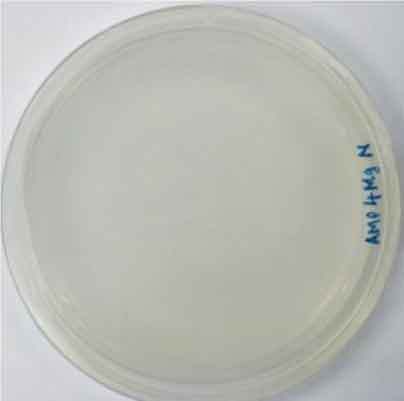 Linear form	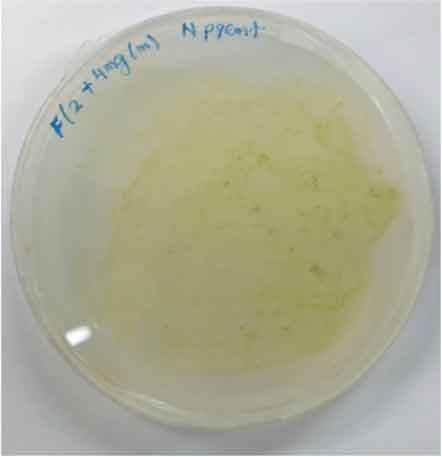 Linear form	
Electroporation with enzyme-treated cells	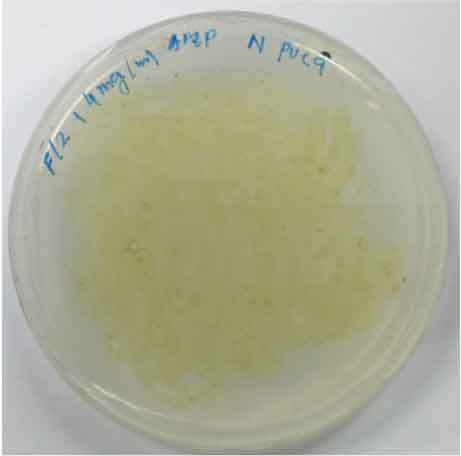 Linear form	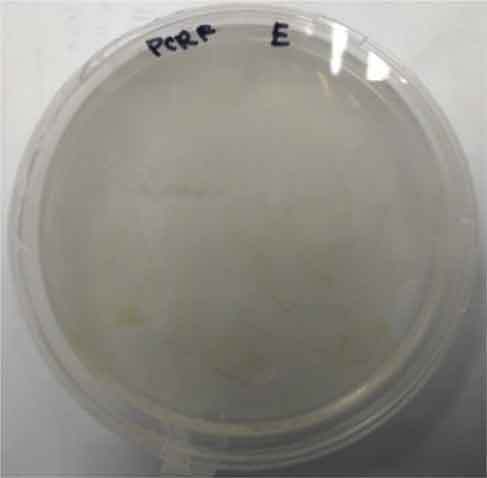 Linear form	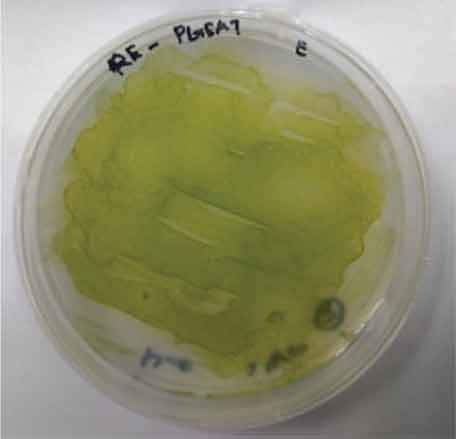 Linear form

**Figure 2.**
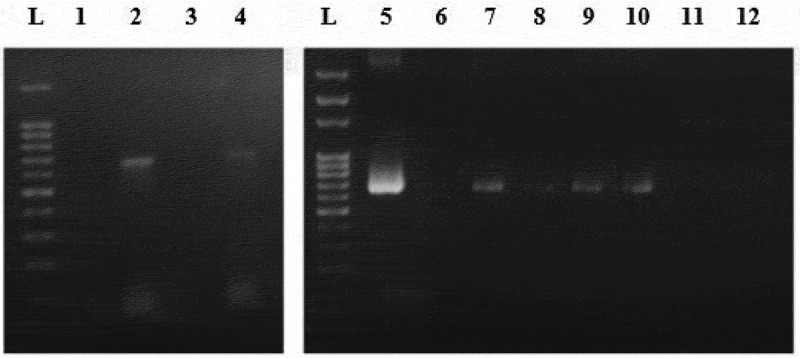
Amplification of AMP^R^ gene fragment from different *Nannochloropsis* sp. transgenic lines genome. Lanes L represent 100bp ladder. Lane 1 represents Wild type. Lane 2 represents circular pUC19 (Electroporation). Lane 4 represents circular pGEM-T (Electroporation). Lane 5 represents Positive control (vector). Lane 6 represents Wild type. Lane 7 represents linear pGEM-T (Electroporation). Lane 8 represents linear pGEM-T (Enzyme-treated cells + Electroporation). Lane 9 represents linear pUC19 (Electroporation). Lane 10 represents linear pUC19 (Enzyme-treated cells + Electroporation). Lane 11 represents PCR fragment (Electroporation). Lane 12 represents PCR fragment (Enzyme-treated cells + Electroporation).

**Figure 3.**
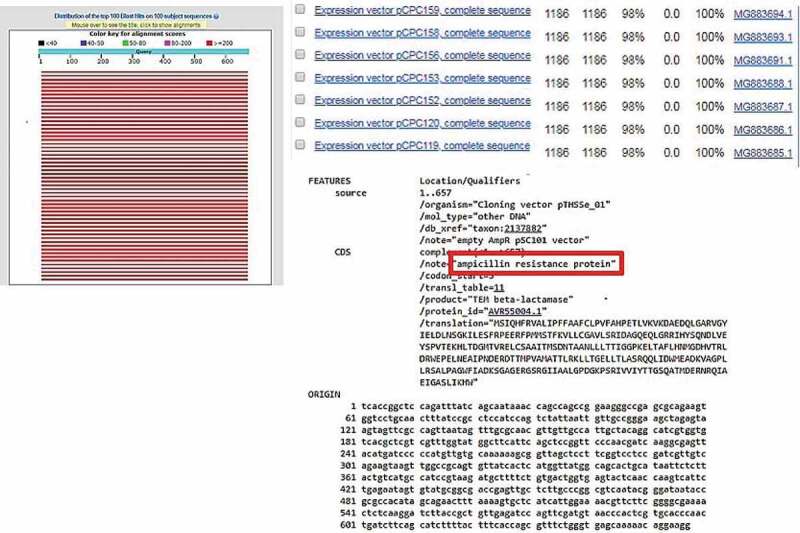
BLAST result of AMP^R^ sequence obtained from sequencing with 100% identity against ampicillin resistance protein.

From this study, only the electroporation method was successfully transformed for non-enzyme-treated *Nannochloropsis* sp. cell using pUC19 and pGEM®-T Easy vector as well as AMP^R^ PCR product. Enzyme-treated cells with electroporation did not manage to uptake the vectors in circular form. In comparison with non-enzyme-treated cells, this might be due to an additional force in introducing with the bulky structure of the plasmid that may disrupt the cell as the circular vector was uptake by the cells without treatment of enzymes and the linear vector was integrated in the cells with enzyme treatment. The cell wall is the basic barrier affecting the transformation efficiency of microalgae. Thus, enzyme-mediated transformation conducted base on the methods of Yang et al. [12 and He et al. [21, with an enzyme mixture of 4% cellulase, 2% macerozyme, and 1% pectinase was used in this study and none of the treated cells yield any transformants, which should result in higher transformation efficiency [12,21]. The inefficiency of the enzyme-mediated transformation probably was due to the further forces used such as high osmotic pressure by the PEG and also the thrust force of the glass beads. Apart from that, negative results from the PEG-mediated results, may be probably due to the absence of carrier DNA in this study. Carrier DNA used to saturate the nucleases present or to block nonspecific DNA-binding sites was generally added to the exogenous DNA in the form of circular plasmids [11]. Twenty-five micrograms of sonicated salmon sperm was used as carrier DNA in the transformation of *Chlorella vulgaris* [12]. However, the glass beads method that used for transformation also yields a negative result for all types of vectors. This result is due to the glass bead size used were too big in comparison of the size of *Nannochloropsis* sp. cells are very small, ranging from 2 to 10 µm [3,22]. Thus, the size of the glass bead utilized in this study is 106 µm which may probably have ruptured the cells when agitated with the cells. Furthermore, no studies have been reported using PEG mediated or glass beads as the method to transform *Nannochloropsis* sp. with any genetic materials. However, stable transformants of *Chlamydomonas reinhardtii* and *Chlorella ellipsoidea* were obtained via glass beads and PEG-mediated methods [23,24]. This study further proves that electroporation is good and may only be the method to genetically modify *Nannochloropsis* sp. For further verification, amplification of AMP^R^ gene fragment from transfected *Nannochloropsis* sp. was carried out. Amplification of AMP^R^ from different transgenic lines of *Nannochloropsis* sp. were found to be positive as shown in Figure 2. No amplification of these genes can be observed in the wild type *Nannochloropsis* sp. For further validation, the PCR products were cloned and sent for sequencing. The obtained sequencing results were analyzed and the BLAST result shows ampicillin-resistant protein as shown in Figure 3. On the other hand, *Nannochloropsis* sp. transfected with PCR product of the AMP^R^ was found to be negative despite it survived the screening process.

Transfected *Nannochloropsis* that survived the screening stage showed positive results for the amplification of the AMP^R^ gene except for the AMP^R^ PCR product transformants. This might be due to the instability of the PCR product integration into the *Nannochloropsis* genome. However, a study by Kilian et al.] produced nitrate or nitrite dependents mutants of *Nannochloropsis* via transformation using PCR product containing a set of genes flanked with homologous sequence to nitrogen reductase in the *Nannochloropsis* genome [7]. In comparison to this study, no flanked of homologous sequence were present in the AMP^R^ PCR product which may lead to a stable integration in the *Nannochloropsis* genome. Overall, the outcome of this study showed that it is possible to transform *Nannochloropsis* sp. using vectors in both linear and circular forms, with different specificities.

## Conclusion

This study intended to determine a stable transformation method for locally isolated *Nannochloropsis* sp via PEG-mediated, electroporation, and glass beads methods. Initially, this study determined the MIC of antibiotics for *Nannochloropsis* sp. This study found that the MIC of ampicillin at 6 mg mL^−1^. In an economic perspective view, the MIC of ampicillin for *Nannochloropsis* sp. was found to be significantly higher compared to other types of antibiotics from previous studies such as hygromycin, zeocin, geneticin, and chloramphenicol. Thus, ampicillin is not a suitable antibiotic for screening due to the high amount requirement which leads to increase in cost for industry or other purposes. This study then proceeds to determine a stable nuclear transformation of Malaysian indigenous species of *Nannochloropsis* sp. However, electroporation was found to be the sole successful method in producing transgenic lines of *Nannochloropsis*. The transformation efficiency can be improved by using lesser concentrations of cell wall degrading enzymes, utilization of carrier DNA, linearized vector, and suitable promoters. The result of this study laid a foundation for further studies involving gene expression in our locally isolated *Nannochloropsis* sp. for various purposes like the delivery of vaccines or other biostimulants to fish via its feed which could greatly reduce the cost in the aquaculture industry.
